# An exploratory study of peripheral joint deterioration and central sensorimotor network reorganization in people with knee osteoarthritis

**DOI:** 10.3389/fbioe.2026.1898313

**Published:** 2026-10-20

**Authors:** Meiping Yang, Qingyang Kang, Cheng Hu, Zibo Gao, Yongjin Li, Dingkun Lin, Yu Hou

**Affiliations:** 1 The Second Clinical Medical College, Guangzhou University of Chinese Medicine, Guangzhou, China; 2 Department of Traditional Chinese Orthopedics and Traumatology, The Third Affiliated Hospital of Southern Medical University, Guangzhou, China; 3 Department of Orthopedics, The Second Affiliated Hospital of Guangzhou University of Chinese Medicine, Guangzhou, China; 4 Guangzhou Key Laboratory of Cervical Mechanobiology, Guangzhou, China; 5 Department of Orthopedics, Guangzhou Orthopedic Hospital, Guangzhou, Guangdong, China

**Keywords:** central sensorimotor network reorganization, fMRI, knee osteoarthritis, proprioception, sEMG

## Abstract

**Introduction:**

This exploratory study aimed to characterize the differential features and intrinsic associations between peripheral proprioceptive deficits, aberrant quadriceps activation, and central sensorimotor network reorganization in patients with knee osteoarthritis (KOA).

**Methods:**

Twenty-eight outpatients with KOA and twenty-eight healthy controls (HC) were prospectively recruited between October 2024 and May 2025. Absolute errors in knee joint position reproduction (JPR) were assessed. Surface electromyography (sEMG) of the quadriceps was recorded during treadmill walking and stair negotiation. Resting-state functional magnetic resonance imaging (rs-fMRI) was employed to characterize spontaneous brain activity and functional connectivity (FC). Intergroup differences were analyzed, and the correlations among these multimodal metrics were evaluated.

**Results:**

Compared with HC, KOA patients exhibited significantly higher JPR errors at 30° and 60°. The RMS of RVM while up and down steps in KOA group had increased activation compared to HC group. Compared with HC group, ALFF increased in cuneus, decreased in superior temporal; fALFF increased in middle Cingulum and middle frontal, decreased in cerebellum; and DC increased in superior parietal and precuneus, but decreased in cerebellum in KOA group. KOA group showed stronger FC between left supplementary motor area and superior frontal; among right paracentral lobule, inferior temporal, middle temporal and superior frontal; among right posterior cingulate cortex, cerebellum, middle frontal and precentral. Correlational Analysis showed that the 45°JPR was negatively correlated with the FC value between SMA_L and superior frontal, the FC value between PoCG _R and middle frontal, respectively. The MDF of LRF in ascending stair was positively correlated with the FC value between SMA_L and superior frontal, the FC value between PoCG _R and middle frontal, respectively.

**Conclusion:**

These exploratory findings suggest a neurophysiological phenotype in KOA characterized by impaired proprioception, compensatory muscle hyperactivity, and central network reorganization. The observed brain-periphery interactions may be associated with​ neurophysiological alterations in KOA pathogenesis, but these results should be interpreted cautiously and require confirmation in larger samples with stricter multiple-comparison correction.

## 1 Introduction

Knee osteoarthritis (KOA) is a prevalent degenerative joint disorder characterized by chronic pain, progressive muscle atrophy, diminished knee extensor strength, joint instability, and restricted range of motion. The decline in motor function in KOA is not solely attributable to peripheral nociceptive sensitization but is also exacerbated by impaired proprioceptive afferents stemming from cartilage degeneration and ligamentous injury. Concurrently, alterations in neuromuscular control and muscle activation patterns contribute substantially to functional deterioration (Hunter and Bierma-Zeinstra, 2019; Yang et al., 2024; Birkbeck et al., 2021; Monteiro et al., 2022). Although traditionally conceptualized as a peripheral joint disease, emerging evidence suggests that KOA is modulated by maladaptive neuroplastic changes within the central nervous system (CNS), including altered pain processing and somatosensory cortical reorganization (Soni et al., 2019; Naumovs et al., 2022; Sohn and Jenei-Lanzl, 2023).

Proprioceptive deficits in KOA represent a multisystem degeneration involving the depletion of mechanoreceptors within degenerated articular tissues, damage to ligamentous proprioceptors, and dysfunction of muscle spindles secondary to disuse atrophy (Salamanna et al., 2023; Khokhlova et al., 2024). Functional MRI (fMRI) studies in related musculoskeletal conditions, such as anterior cruciate ligament reconstruction, have linked proprioceptive inaccuracies to hyperactivation in the anterior cingulate gyrus and insular cortex (Strong et al., 2022).

Aberrant quadriceps activation patterns, particularly in the vastus medialis, are hallmark features of KOA-related functional decline (Chen et al., 2023; Liu et al., 2025). Manifestations include delayed onset of activation during gait, increased co-contraction of antagonist muscles, and premature muscle fatigue, likely representing compensatory mechanisms for mechanical instability (Chen et al., 2023; Yamauchi et al., 2020; Cunha et al., 2019; Rashid et al., 2022; Taniguchi et al., 2024).

Recent neuroimaging studies have further mapped structural and functional brain changes in KOA. For instance, Kang et al. (2022) reported a correlation between gray matter atrophy in the left middle temporal gyrus and clinical functional scores, suggesting a link between structural degeneration and pain modulation. Moreover, Shanahan et al. (2015) demonstrated a significant anterior displacement of the motor cortical representation of the knee in KOA patients, indicating a distorted somatotopic organization that may underlie motor deficits. Despite these advances, the integrative relationship between peripheral proprioceptive impairment, quadriceps dysfunction, and central network reorganization remains incompletely understood. We hypothesized that KOA progression follows a maladaptive cascade involving “peripheral sensory distortion-muscle regulatory failure-central network remodeling.” This exploratory study aims to bridge these domains by integrating biomechanical assessments with resting-state fMRI to elucidate the “brain-joint” interplay in KOA.

## 2 Methods

### 2.1 Participants

Participants for this cross-sectional study were recruited from October 2024 to May 2025 using convenience sampling at orthopedic clinics, social media, and referrals. A total of 30 patients with knee osteoarthritis (KOA) and 30 healthy controls (HC) matched for age, height, and weight, were enrolled. The inclusion criteria for KOA patients are as followed: aged 55–75 years, right-handedness, average knee pain ≤5 on a 10-point Visual Analog Scale (VAS), and Kellgren-Lawrence (K-L) radiographic grade 3 or 4. HC had no history of knee pain or injury within the prior 5 years. Exclusion criteria for all participants: contraindications to MRI, history of neurological/psychiatric disorders, orthopedic surgery, systemic diseases affecting mobility, or excessive head motion. Two KOA patients and two HC were excluded from the final analysis: two due to intolerance to the MRI environment, and two due to excessive head motion (>2.5 mm translation or >2.5° rotation). The final cohort included 28 KOA patients (all female) and 28 HC (25 females, 3 males). This study was approved by the Clinical Trial Ethics Committee of the Guangdong Hospital of Traditional Chinese Medicine. Written informed consent was obtained from all participants prior to enrollment.

### 2.2 Imaging data acquisition

All participants underwent MRI scanning on a 3.0T MRI scanner (Magnetom Verio, Siemens, Erlangen, Germany) equipped with a 32-channel head coil at the Guangdong Hospital of Traditional Chinese Medicine. A high-resolution three-dimension sagittal magnetizationprepared rapid acquisition gradient echo (MPRAGE) T1-weighted sequence was obtained with the following parameters: 128 slices, TR of 8,100 m, TE of 3.1 m, slice thickness of 1 mm, FA of 8°, matrix size of 256 × 256, field of view (FOV) of 256 × 256 mm^2^. The resting-state functional images were acquired using a gradient echo planar imaging sequence with the following parameters: 43 contiguous axial slices, repetition time (TR) of 2,000 m, echo time (TE) of 30 m, slice thickness of 3.2 mm, flip angle (FA) of 90°, matrix size of 64 × 64, field of view (FOV) of 200 × 200 mm^2^, total scan time of 8 min. During the rsfMRI scan, all the participants were given no task but were instructed to simply rest with eyes closed.

### 2.3 rs-fMRI preprocessing

The rs-fMRI data were processed using SPM12 and RESTplus V1.31 (Jia et al., 2019). The data preprocessing included the following steps: (a) discarding the first 10 time points of each participant to avoid magnetic saturation effects and for participants to adapt to the scanning noise; (b) slice timing; (c) head motion correction (subjects with more than 2.5 mm displacement in any of x, y, and z directions or 2.5°of angular rotation were removed); (d) co-registration, spatial normalization (an individual T1-weighted image was co-registered to the mean functional image and then the T1-weighted image was segmented into gray matter, white matter signal, cerebrospinal fluid signal, soft tissue, skull and air), and resampling to 3 × 3 × 3 mm resolution; (e) spatial smoothing with an isotropic Gaussian kernel at full width and half maximum (FWHM) of 6 mm; (f) removing the linear trend and regressing nuisance covariates, including the Friston-24 head motion parameters (Friston et al., 1996), signals of white matter and cerebrospinal fluid (CSF).

### 2.4 Static ALFF, fractional ALFF (fALFF), DC and FC calculation

We performed ALFF, fALFF, DC and FC analysis for preprocessed fMRI data. The calculation of ALFF was based on fast Fourier transform (FFT). Using FFT, each time course was converted to the frequency domain. Then, the square root of the power spectrum at each frequency was averaged across the filtered band (0.01–0.08 Hz). The ALFF of each voxel was then normalized by the global mean of the ALFF values (mALFF) for standardization. For each given voxel, mALFF reflected the degree of its raw ALFF value relative to the average ALFF value of the whole brain (Zang et al., 2007). Then we calculated fALFF by obtaining the ratio of the power spectrum of low frequency (0.01–0.08 Hz) to that of the entire frequency range. Then, the resulting spatial fALFF maps were then divided by each voxel divided by the whole brain fALFF mean (mfALFF), providing mfALFF spatial maps (Zou et al., 2008). DC was calculated as the number or weighted sum of significant connections between a voxel’s time course with other voxels’ time course (Buckner et al., 2009; Zuo et al., 2012). In this study, weighted DC was used as it could provide a more precise centrality characterization of functional brain networks than binary DC (Luo et al., 2020). We restricted analysis to positive correlations above a threshold of r = 0.25 to eliminate voxels with weak temporal correlations caused by noise or white matter. For FC calculation, according to the research results of previous literature, relevant brain regions (precentral gyrus, paracentral lobule and postcentral gyrus) (Shanahan et al., 2015; Smith et al., 2023) differentiating KOA patients and health controls were selected as seeds. Spherical seeds were created using the peak Montreal Neurological Institute (MNI) coordinates as shown in Table 1. A Pearson’s correlation analysis was then performed between each seed’s mean time course and each voxel’s time course in the rest of the brain. Finally, the resulting correlation coefficients were normalized into Z values using Fisher’s R-to-Z transformation.

**TABLE 1:** The MNI coordinates of seeds.

Region name	Side	x	y	z	Radius (mm)
Supplementary motor area (SMA)	Left	−4	4	52	6
Paracentral lobule (PCL)	Right	8	−28	64	6
Postcentral gyrus (PoCG)	Right	12	−34	68	6

### 2.5 Absolute error angle of joint position reproduction (JPR)

The YTK-E continuous passive motion (CPM) device was applied. Testing was performed on the affected knee for KOA patients (right knee for 15 patients, left knee for 13 patients) and the right (dominant) knee for HC. Participants were blindfolded and instructed to relax. The knee was passively flexed at 2°·s^-1^ to target angles (30°, 45°, 60°), held for 5 s, and returned to 0°. Participants then actively replicated the perceived angle. Three trials were conducted per angle, and the absolute angular error of JPR was calculated as the mean deviation from the target. Prior to formal testing, all participants completed one familiarization trial to minimize procedural error. Intraclass Correlation Coefficients (ICC) for test-retest reliability were calculated using a two-way random-effects model. Smaller absolute errors indicated superior proprioceptive accuracy (Salamanna et al., 2023; Khokhlova et al., 2024; Strong et al., 2022).

### 2.6 Surface electromyography (sEMG) data of the quadriceps muscle

The 8-channel distributed wireless electromyography acquisition system (Shenzhen Runyi Taiyi Technology Co., Ltd.), featuring a sampling frequency of 2000 Hz and A three-dimensional motion capture gait system (Opti_Knee, Innomotion Inc., Shanghai, China) were employed for data collection. The electrodes were positioned at the highest point of the muscle bellies of the assessed muscles (bilateral vastus lateralis, rectus femoris, and vastus medialis), aligned with the orientation of the muscle fibers. Collect Maximum Voluntary Contraction (MVC) data in accordance with standardized procedures. Subsequently, participants engaged in treadmill walking, modifying the speed between 1 km/h and 3 km/h to align with their customary walking rates, after which they ascended and descended the steps three times. The sEMG measurements of the quadriceps muscle were acquired during these movements. MATLAB analysis software was employed to process the original sEMG signals from the vastus medialis and vastus lateralis. The original sEMG signals are subjected to denoising *via* a 20–450 Hz bandpass Butterworth filter, followed by rectification and smoothing for preprocessing. The phase for walking we analyzed was the Support phase of the gait cycle (0%–60%). In the task of ascending and descending stairs, the limb that initiates the movement on the stairs is defined as the leading phase, while the limb that follows subsequently is referred to as the trailing phase. MATLAB analysis software was then employed to derive the amplitude values for all exercise cycles, determine the time range of each signal cycle based on the raw signals, and identify the Root Mean Square (RMS) and the Median Frequency (MDF) for each muscle of both left and right knee within the Support phase of the gait cycle (0%–60%) and leading phase. Additionally, the RMS were normalized [RMS (walking or up stairs)/RMS(MVC)] using data from the MVC test.

### 2.7 Statistical analysis

Sample size estimation was guided by prior fMRI studies on KOA, which indicate that 20–25 participants per group provide adequate statistical power to detect medium-to-large effect sizes (Kang et al., 2022). Considering potential dropout due to excessive head motion, we recruited 30 participants per group. Demographic and clinical characteristics were summarized as mean ± standard deviation (SD) or frequencies (%). Group differences for continuous variables were assessed using two-sample *t*-tests for normally distributed data and Mann-Whitney U tests for non-normally distributed data. ICC for JPR was calculated using a two-way random-effects model. For rs-fMRI data, voxel-wise two-sample *t*-tests were performed using DPABI (Yan et al., 2016), incorporating age and education as covariates. To control for multiple comparisons, Gaussian Random Field (GRF) theory correction was applied: the primary analysis used a voxel-level threshold of *p* < 0.05 and cluster-level threshold of *p* < 0.05, and a secondary analysis using a more stringent voxel-level threshold of *p* < 0.001 (with GRF cluster *p* < 0.05) was also performed (Yan et al., 2016). Mean framewise displacement (FD) was 0.08 ± 0.02 mm for the KOA group and 0.07 ± 0.02 mm for HC (*p* = 0.21), confirming minimal motion-related confounding.

Partial correlation analyses (SPSS v27.0; IBM, Armonk, NY, USA) were utilized to examine associations between biomechanical variables (JPR, sEMG) and rs-fMRI metrics, adjusting for age in Benjamini-Hochberg False Discovery Rate (FDR) correction. Statistical significance was set at *p* < 0.05. The target FDR was set at *q* < 0.05. Adjusted p-values (*q*-values) are reported alongside uncorrected p-values, Effect sizes (*r*) and 95% confidence intervals for all significant correlations.​

## 3 Results

### 3.1 Basic characteristics

Demographic and clinical characteristics of KOA group (28 patients, all female; mean age, 64.17 ± 5.33 years) and HC group (28 subjects, 25 women and 3 men; mean age, 61.40 ± 4.88) are listed in Table 2. There were no significant differences between the two groups in terms of gender, BMI, educational level, and fMRI scan head movement data (*p* > 0.05). Detailed demographics and the psychological characteristics of the two groups are shown in Table 2.

**TABLE 2:** Details of characteristics between KOA group and HC group.

Variable	KOA group (n = 28) (mean ± std)	HC group (n = 28) (mean ± std)	*p*-value
Age (years)	64.17 ± 5.33	61.40 ± 4.88	0.05
Gender (F/M)	28/0	25/3	0.24
BMI (kg/m^2^)	22.92 ± 3.21	22.50 ± 3.50	0.86
Education level (years)	13.50 ± 3.03	13.95 ± 2.31	0.74
Affected knee (right/left)	15/13	—	—
K- L grade (III/IV grade)	11/17	—	—
VAS score	1.96 ± 1.17	—	—
WOMAC score	8.43 ± 7.43	—	—
30°JPR	5.82 ± 5.18	3.75 ± 2.57	0.03*
45°JPR	8.55 ± 5.24	6.65 ± 4.46	0.15
60°JPR	9.82 ± 5.23	6.92 ± 5.04	0.02*
Walk_LVM_RMS(MVC)	1.36 ± 1.61	0.84 ± 0.49	0.12
Walk_LRF_RMS(MVC)	0.76 ± 0.33	0.92 ± 0.61	0.72
Walk_LVL_RMS(MVC)	0.73 ± 0.38	0.63 ± 0.25	0.40
Walk_RVM_RMS(MVC)	1.32 ± 0.94	1.10 ± 0.89	0.12
Walk_RRF_RMS(MVC)	0.95 ± 0.42	0.96 ± 0.66	0.60
Walk_RVL_RMS(MVC)	0.80 ± 0.43	0.74 ± 0.41	0.42
Stair_LVM_RMS(MVC)	1.15 ± 0.29	1.12 ± 0.57	0.25
Stair_LRF_RMS(MVC)	1.02 ± 0.42	0.93 ± 0.27	0.35
Stair_LVL_RMS(MVC)	0.99 ± 0.23	0.86 ± 0.30	0.09
Stair_RVM_RMS(MVC)	1.20 ± 0.47	0.98 ± 0.52	0.02*
Stair_RRF_RMS(MVC)	1.31 ± 1.52	0.90 ± 0.39	0.10
Stair_RVL_RMS(MVC)	0.89 ± 0.43	0.78 ± 0.29	0.33
Walk_LVM_MDF	43.71 ± 15.39	41.67 ± 15.59	0.62
Walk_LRF_MDF	50.50 ± 16.41	48.78 ± 14.44	0.68
Walk_LVL_MDF	55.79 ± 17.63	56.33 ± 16.45	0.91
Walk_RVM_MDF	53.07 ± 21.33	50.99 ± 26.43	0.75
Walk_RRF_MDF	50.69 ± 15.00	52.04 ± 15.81	0.74
Walk_RVL_MDF	61.45 ± 11.35	55.86 ± 16.34	0.14
Stair_LVM_MDF	51.21 ± 12.95	53.46 ± 13.66	0.53
Stair_LRF_MDF	57.49 ± 11.84	58.57 ± 14.95	0.77
Stair_LVL_MDF	59.51 ± 16.41	61.10 ± 12.59	0.69
Stair_RVM_MDF	53.17 ± 25.86	59.46 ± 21.80	0.33
Stair_RRF_MDF	56.52 ± 16.85	58.27 ± 15.25	0.69
Stair_RVL_MDF	62.44 ± 10.94	61.64 ± 11.34	0.79

BMI, body mass index; K-L = kellgren and lawrence; VAS, visual analog scale; WOMAC, Western Ontario and McMaster Universities Arthritis Index; JPR, joint position reproduction; LVM, left vastus medialis; LRF, left rectus femoris; LVL, left lateral femoral; RVM, right vastus medialis; RRF, right rectus femoris; RVL, right vastus lateralis; RMS, root mean square; MDF, median frequency; MVC, Maximum Voluntary Contraction. Normality was assessed using the Shapiro-Wilk test. Normally distributed data were compared using independent samples t-tests; non-normally distributed data were compared using the Mann-Whitney U test.

^*^ = *p* < 0.05.

### 3.2 Absolute Error Angles in JPR

The ICC for the repeated tests at knee flexion angles of 30°, 45°, and 60° were 0.696, 0.794, and 0.772, respectively, demonstrating a significant correlation (*p* < 0.05). KOA patients showed significantly larger JPR errors at 30° and 60° compared to HC (*p* < 0.05). No significant intergroup difference was observed at 45°.

### 3.3 Quadriceps sEMG in walking and walking up and down steps

The signal of LVM, LRF, LVL, RVM, RRF and RVL in walking and walking up and down steps was collected and processed. As shown in Table 2, the RMS of RVM while up and down steps in KOA group had increased activation compared to HC group.

### 3.4 The comparison of local rsfMRI metrics

As shown in Figure 1, compared with HC group, ALFF increased in cuneus, but decreased in superior temporal in KOA group (Table 3; Figure 1a); fALFF increased in middle cingulum and middle frontal, but decreased in cerebellum (Table 3; Figure 1b); DC weighted increased in superior parietal and precuneus, but decreased in cerebellum (Table 3; Figure 1c), after GRF correction with a voxel-level threshold of *p* < 0.05 and a cluster-level threshold of *p* < 0.05. No significant clusters between two groups after GRF correction with a voxel-level threshold of *p* < 0.001.

**FIGURE 1:**
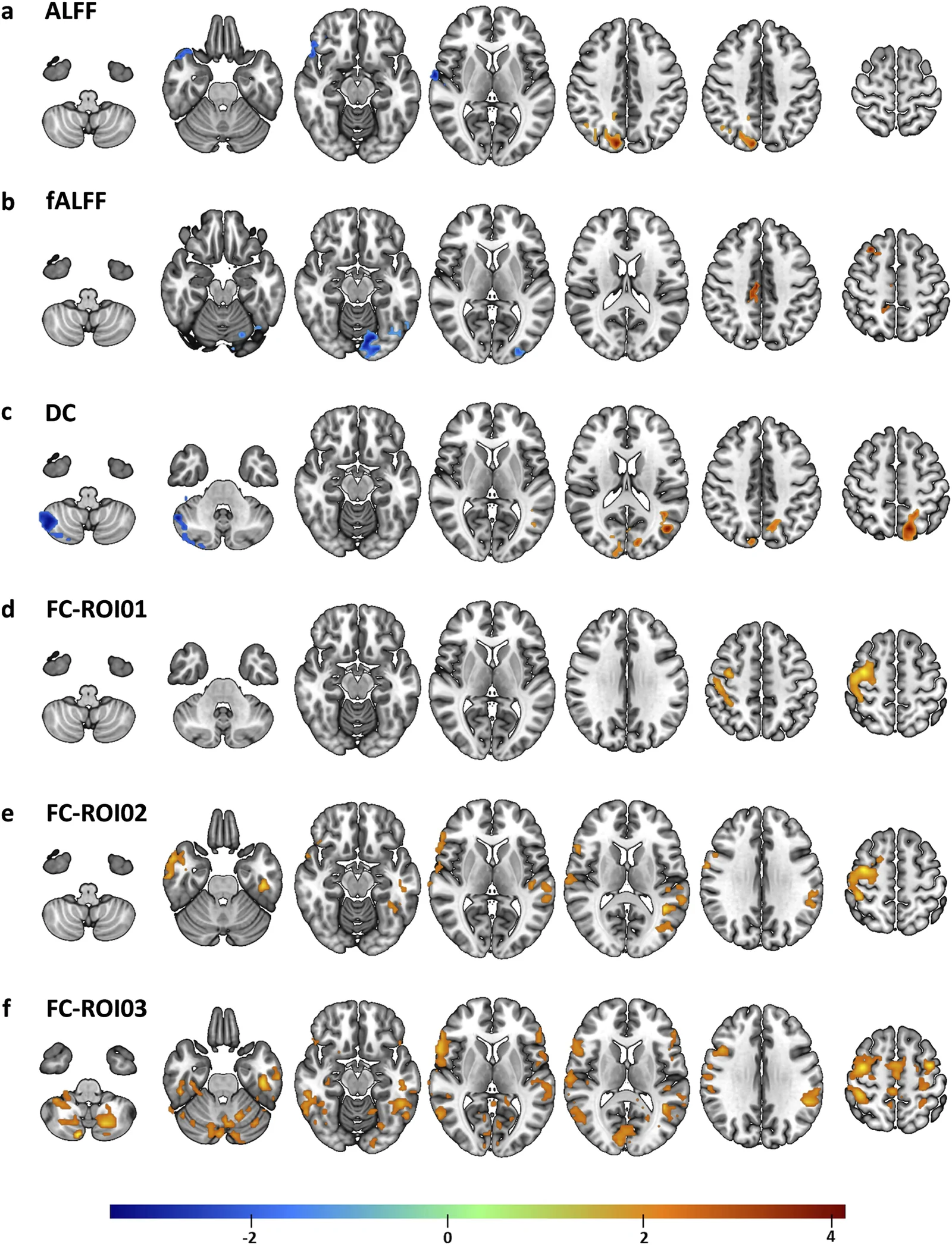
Brain regions showing different rsfMRI values between the KOA and HC group. **(a)** Brain regions with significant differences in ALFF between the KOA and HC group. **(b)** Brain regions with significant differences in fALFF between the KOA and HC group. **(c)** Brain regions with significant differences in DC between the KOA and HC group. **(d)** Brain regions with significant differences in FC-ROI01 between the KOA and HC group. **(e)** Brain regions with significant differences in FC-ROI02 between the KOA and HC group. **(f)** Brain regions with significant differences in FC-ROI03 between the KOA and HC group. The warm colors indicating increased value and cool colors indicating decreased value in the KOA compared with HC group. (after GRF correction; voxel-wise *p* < 0.05, cluster-wise *p* < 0.05, two-tailed). The color bar indicates the T-value.

**TABLE 3:** Brain regions with significantly different rsfMRI values in the KOA group compared with the HC group.

​	​	​	​	Peak MNI coordinates			​	​
	Cluster	Brain region	Side	x	y	z	Cluster size	Peak value
ALFF								
​	Cluster 1	Temporal_Sup	Right	66	−3	6	197	−3.96
​	Cluster 2	Cuneus	Right	12	−81	39	350	4.69
fALFF								
​	Cluster1	Cerebellum_6	Left	−18	−78	−15	274	−4.59
​	Cluster 2	Cingulum_Mid	Right	6	−18	42	211	3.96
​	Cluster 3	Frontal_Mid	Right	30	24	54	251	3.65
smDC_weighted								
​	Cluster1	Cerebellum_Crus1	Right	51	−57	−36	461	−3.53
​	Cluster 2	Parietal_Sup	Left	−15	−69	54	773	4.33
ROI-1(FC-sma_l)								
​	Cluster 1	Frontal_Sup	Right	36	−6	57	551	4.21
ROI-2(FC-PCL_R)								
​	Cluster1	Temporal_Inf	Right	60	−12	−27	482	3.55
​	Cluster 2	Temporal_Mid	Left	−42	−51	12	605	4.28
​	Cluster 3	Frontal_Sup	Right	36	−6	57	435	4.52
ROI-3(FC- PoCG _R)								
​	Cluster1	Cerebellum_Crus2	Right	12	−84	−45	2736	5.00
​	Cluster 2	Frontal_Mid	Right	39	−6	57	2097	4.99
​	Cluster 3	Precentral	Left	−39	−3	57	541	4.92

### 3.5 The comparison of FC

The results of seed-based FC analyzes are shown in Table 3 and Figures 1d-f. Compared to HC participants, for the seed1 (left supplementary motor area, SMA_L), KOA patients showed higher FC with superior frontal, for the seed2 (right paracentral lobule, PCL_R), KOA patients showed higher FC with inferior temporal, middle temporal and superior frontal, and for the seed3 (right postcentral gyrus, PoCG _R), KOA patients showed higher FC with cerebellum, middle frontal and precentral, after GRF correction with a voxel-level threshold of *p* < 0.05 and a cluster-level threshold of *p* < 0.05. No significant clusters between two groups after GRF correction with a voxel-level threshold of *p* < 0.001.

### 3.6 Partial Correlational Analysis

The partial correlation between the Absolute Error Angles in JPR, sEMG of the quadriceps muscle and rsfMRI metrics in the KOA group is detailed in Figure 2. The 45°JPR was negatively correlated with the FC value between SMA_L and superior frontal (*r* = −0.440, 95% *CI* [-0.693, −0.087], *p* = 0.022, *q* = 0.066), the FC value between PoCG _R and middle frontal (*r* = −0.540, 95% *CI* [-0.750, −0.274], *p* = 0.002, *q* = 0.021), respectively. The MDF of LRF in ascending stair was positively correlated with the FC value between SMA_L and superior frontal (*r* = 0.415, 95% *CI* [0.088,0.651 ], *p* = 0.031, *q* = 0.072),the FC value between PoCG _R and middle frontal (*r* = 0.469, 95% *CI* [0.184, 0.687], *p* = 0.013, *q* = 0.068), respectively.

**FIGURE 2:**
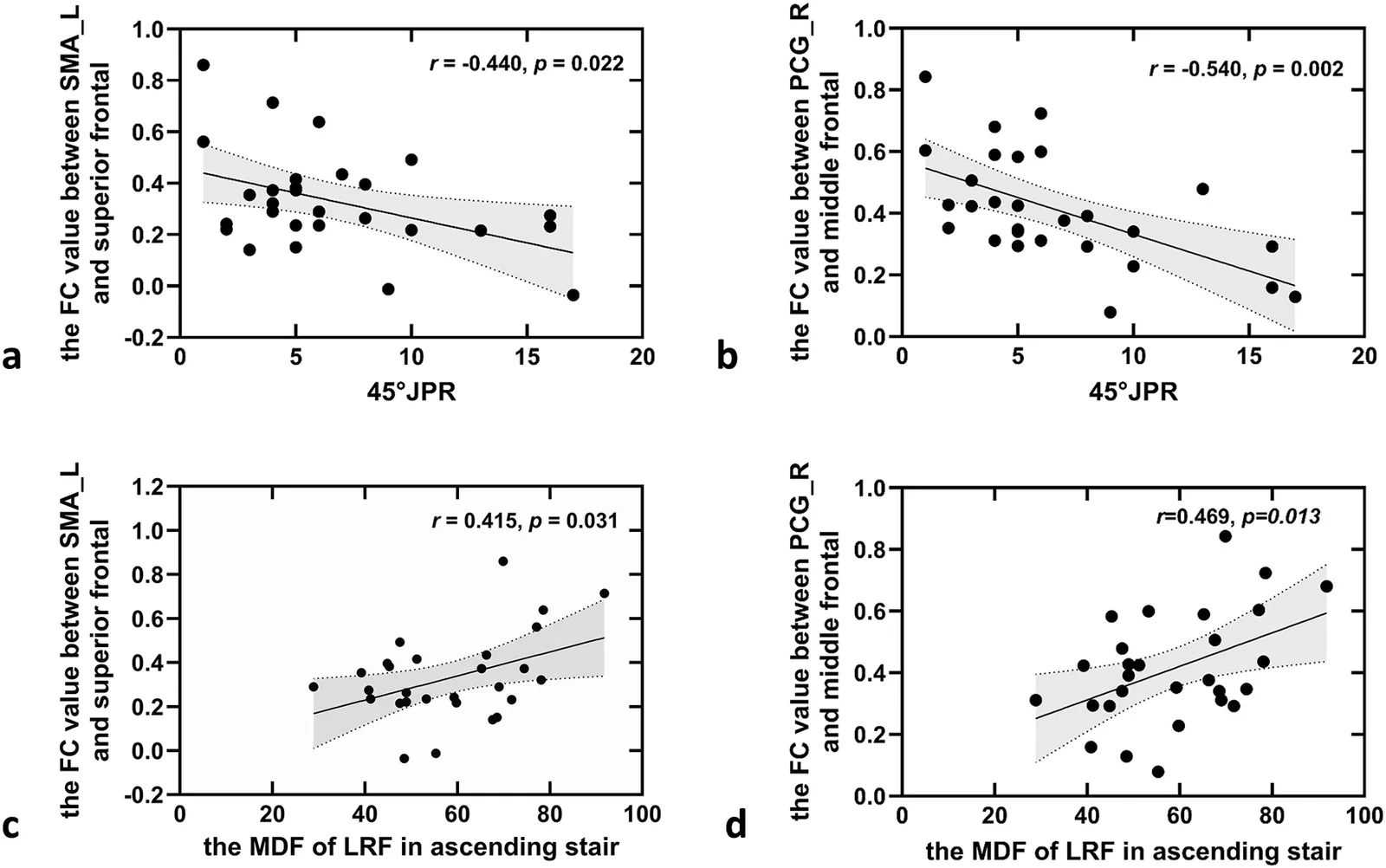
The partial correlation between the Absolute Error Angles in JPR, sEMG of the quadriceps muscle and rsfMRI metrics in the KOA group. **(a)** The correlation in 45°JPR and the FC value between SMA_L and superior frontal; **(b)** The correlation in 45°JPR and the FC value between PoCG_R and middle frontal; **(c)** The correlation in MDF of LRF in ascending stair and the FC value between SMA_L and superior frontal; **(d)** The correlation in MDF of LRF in ascending stair and the FC value between PoCG _R and middle frontal.

## 4 Discussion

This study provides a comprehensive multimodal characterization of the neurophysiological alterations in KOA, integrating peripheral biomechanical deficits with central network reorganization. Our findings substantiate that KOA is not merely a degenerative joint disorder but a systemic condition involving maladaptive neuroplasticity across the sensorimotor continuum.

Consistent with prior literature, we observed significant proprioceptive inaccuracies at 30° and 60° of knee flexion in KOA patients (Yang et al., 2024; Khokhlova et al., 2024; Zeng et al., 2022). These deficits likely arise from the degradation of intra-articular mechanoreceptors (Salamanna et al., 2023). Interestingly, while group differences were most pronounced at 30° and 60°, the 45° JPR—despite showing no intergroup difference—exhibited the most robust correlations with brain connectivity. This suggests that subtle, subclinical variations in proprioceptive acuity may serve as sensitive proxies for underlying central network integrity. The sEMG findings revealed compensatory hyperactivity, elevated RMS, of the vastus medialis during dynamic tasks, reflecting a biomechanical adaptation to enhance joint stability amid muscle weakness and laxity (Chen et al., 2023; Taniguchi et al., 2024; Pan et al., 2023; Hansen et al., 2024). Meanwhile, MVC normalization was performed per muscle, the normalized values >1 can occur during dynamic functional tasks, such as going up stair, where muscle activation briefly exceeds the isometric MVC recorded in a laboratory setting, particularly in patients compensating for weakness. The task-dependent nature of these alterations—restricted to specific angles and functional tasks—highlights the context-specificity of neuromuscular adaptation in KOA, wherein impaired joint afferents disrupt proprioceptive-motor integration required for stable locomotion (Khokhlova et al., 2024; Cunha et al., 2019; Rashid et al., 2022).

The neuroimaging results delineate a profile of central reorganization. Increased ALFF and fALFF in the cuneus and frontal regions may reflect heightened attentional resource allocation and cognitive monitoring of knee mechanics (Kang et al., 2022). Conversely, decreased activity in the cerebellum and temporal lobes suggests compromised sensorimotor integration. The observed hyperconnectivity involving the SMA, PoCG, and frontal-parietal circuits likely represents an adaptive, albeit inefficient, neural strategy to compensate for degraded peripheral afferent input (Mallio et al., 2022; Li et al., 2025). A notable phenotypic feature of our cohort was the discordance between advanced radiographic severity with III-IV K-L grade and relatively low symptom burden with low VAS or WOMAC scores. This “structural-severe but symptomatic-mild” phenotype may reflect a subgroup with effective central compensatory mechanisms or high pain tolerance. Consequently, the central alterations observed here might be driven more by structural joint degeneration than by acute nociceptive input, a distinction that warrants consideration in future stratified KOA research.

Partial correlation analyses, controlling for age within the KOA group, revealed several associations between proprioceptive accuracy, quadriceps sEMG parameters, and rs-fMRI functional connectivity. After FDR correction for multiple comparisons across the full set of tests, one association remained significant: the 45°JPR error was negatively correlated with the functional connectivity between the right PoCG_R and the middle frontal gyrus. This robust inverse association indicates that stronger intrinsic functional integration between the primary somatosensory cortex and prefrontal executive regions is linked to better preservation of proprioceptive acuity in KOA. The middle frontal gyrus is critically involved in sensorimotor integration and attentional modulation of somatosensory processing; thus, enhanced PoCG–middle frontal connectivity may represent a compensatory mechanism that helps maintain joint position sense despite degenerative changes in the knee (Tang et al., 2025). In addition, three associations were nominally significant but did not survive FDR correction. The 45°JPR error was negatively correlated with the functional connectivity between the SMA_L and the superior frontal gyrus, suggesting a potential role of premotor–prefrontal networks in proprioceptive calibration, albeit this finding requires cautious interpretation given the lack of strict statistical significance after correction. Similarly, the MDF of the LRF during stair ascent was positively correlated with functional connectivity of both the SMA_L–superior frontal network and the PoCG_R–middle frontal network. These nominal associations imply that preserved sensorimotor network integrity may support maintenance of muscle fiber conduction velocity and fatigue resistance in the quadriceps, highlighting a potential protective role of intact central somatosensory function against progressive neuromuscular deterioration in KOA. However, the failure to survive FDR correction underscores the need for replication in larger samples. Collectively, these findings substantiate the functional relevance of somatosensory and frontoparietal network reorganization to peripheral impairment in KOA. The consistent involvement of PoCG_R–middle frontal connectivity across both proprioceptive and sEMG measures points to a central compensatory cascade wherein prefrontal resources are recruited to augment impaired somatosensory processing, with variable success in offsetting peripheral neuromuscular decline.

## 5 Limitation

Several limitations warrant acknowledgment. First, the all-female KOA cohort limits generalizability to male patients. Second, the cross-sectional design precludes causal inferences regarding the temporal sequence of brain-periphery interactions. Third, simultaneous sEMG and fMRI data could not be acquired due to electromagnetic interference from the MRI environment, precluding real-time correlation of muscle activation with hemodynamic responses. Fourth, while JPR is a validated clinical proxy for proprioception, it remains an indirect behavioral measure; ICC values for JPR ranged from 0.696 to 0.794 (moderate-to-good reliability), and future studies should use quantitative sensory testing or microneurography for more precise afferent signaling assessment. Finally, the “structural-severe but symptomatic-mild” phenotype of our cohort may limit extrapolation of findings to KOA patients with high pain burden.

## 6 Conclusion

In conclusion, KOA is associated with a complex neurophysiological phenotype characterized by impaired proprioception, compensatory increases in muscle activation amplitude, and significant reorganization of large-scale brain networks. These alterations highlight a dynamic interplay between peripheral joint deterioration and central nervous system adaptation. The significant associations between specific biomechanical parameters and intrinsic brain connectivity underscore the existence of a tightly coupled brain-joint interface. These findings advocate for a paradigm shift from purely biomechanical models of KOA toward comprehensive neuro-biomechanical frameworks. Future therapeutic strategies should consider integrating interventions targeting central adaptations—such as neuromuscular retraining and sensory integration therapies—alongside peripheral strengthening regimens to effectively restore function and mitigate disability.

## Data Availability

The datasets presented in this study can be found in online repositories. The names of the repository/repositories and accession number(s) can be found in the article/Supplementary Material.

## References

[B1] Birkbeck M. G. Blamire A. M. Whittaker R. G. Sayer A. A. Dodds R. M. (2021). The role of novel motor unit magnetic resonance imaging to investigate motor unit activity in ageing skeletal muscle. J. Cachexia Sarcopenia Muscle 12 (1), 17–29. 10.1002/jcsm.12655 33354940 PMC7890268

[B2] Buckner R. L. Sepulcre J. Talukdar T. Krienen F. M. Liu H. Hedden T. (2009). Cortical hubs revealed by intrinsic functional connectivity: mapping, assessment of stability, and relation to Alzheimer's disease. J. Neurosci. 29 (6), 1860–1873. 10.1523/JNEUROSCI.5062-08.2009 19211893 PMC2750039

[B3] Chen W. Li C. Wang Y. Shen X. Wu Z. Li J. (2023). Comparison of the asymmetries in muscle mass, biomechanical property and muscle activation asymmetry of quadriceps femoris between patients with unilateral and bilateral knee osteoarthritis. Front. Physiol. 14, 1126116. 10.3389/fphys.2023.1126116 37284540 PMC10239935

[B4] Cunha J. E. Barbosa G. M. Castro PATS Luiz B. L. F. Silva A. C. A. Russo T. L. (2019). Knee osteoarthritis induces atrophy and neuromuscular junction remodeling in the quadriceps and tibialis anterior muscles of rats. Sci. Rep. 9 (1), 6366. 10.1038/s41598-019-42546-7 31019213 PMC6482306

[B5] Friston K. J. Williams S. Howard R. Frackowiak R. S. Turner R. (1996). Movement-related effects in fMRI time-series. Magn. Reson Med. 35 (3), 346–355. 10.1002/mrm.1910350312 8699946

[B6] Hansen L. Rogoschin J. Komnik I. (2024). Muscle synergies in patients with medial knee osteoarthritis during Level-Ramp- and stair locomotion. J. Mot. Behav. 3, 1–11. 10.1080/00222895.2024.2435829 39626885

[B7] Hunter D. J. Bierma-Zeinstra S. (2019). Osteoarthritis. Lancet 393 (10182), 1745–1759. 10.1016/S0140-6736(19)30417-9 31034380

[B8] Jia X. Z. Wang J. Sun H. Y. Zhang H. Liao W. (2019). RESTplus: an improved toolkit for resting-state functional magnetic resonance imaging data processing. Sci. Bull. (Beijing) 64 (14), 953–954. 10.1016/j.scib.2019.05.008 36659803

[B9] Kang B. X. Ma J. Shen J. Xu H. Wang H. Q. Zhao C. (2022). Altered brain activity in end-stage knee osteoarthritis revealed by resting-state functional magnetic resonance imaging. Brain Behav. 12 (1), e2479. Epub 2021 Dec 29. 10.1002/brb3.2479 34967156 PMC8785636

[B10] Khokhlova L. Komaris D. S. O'Flynn B. Tedesco S. (2024). Correlation between proprioception, functionality, patient-reported knee condition and joint acoustic emissions. PLoS One 19 (11), e0310123. 10.1371/journal.pone.0310123 39504329 PMC11540232

[B11] Li C. Hao C. Zhou K. Zhang P. Sun Q. Li Z. (2025). Inflammation-mediated regional brain alterations associated with mild cognitive impairment in knee osteoarthritis. Arthritis Res. Ther. 27 (1), 181. 10.1186/s13075-025-03646-0 41013635 PMC12465908

[B12] Liu S. Du Z. Song L. Liu H. Tee C. A. T. H. Liu H. (2025). Factors, characteristics and influences of the changes of muscle activation patterns for patients with knee osteoarthritis: a review. J. Orthop. Surg. Res. 20 (1), 112. 10.1186/s13018-025-05484-x 39885604 PMC11780770

[B13] Luo F. F. Xu H. Zhang M. Wang Y. (2020). Abnormal regional spontaneous brain activity and its indirect effect on spasm ratings in patients with hemifacial spasm. Front. Neurosci. 14, 601088. 10.3389/fnins.2020.601088 33362459 PMC7756088

[B14] Mallio C. A. Bernetti C. Agostini F. Mangone M. Paoloni M. Santilli G. (2022). Advanced MR imaging for knee osteoarthritis: a review on local and brain effects. Diagn. (Basel) 13 (1), 54. 10.3390/diagnostics13010054 36611346 PMC9818324

[B15] Monteiro T. S. King B. R. Seer C. Mantini D. Swinnen S. P. (2022). Network-specific differences in transient brain activity at rest are associated with age-related reductions in motor performance. Neuroimage 252, 119025. 10.1016/j.neuroimage.2022.119025 35202812

[B16] Naumovs V. Groma V. Mednieks J. (2022). From low-grade inflammation in osteoarthritis to neuropsychiatric sequelae: a narrative review. Int. J. Mol. Sci. 23 (24), 16031. 10.3390/ijms232416031 36555670 PMC9784931

[B17] Pan J. Huang W. Huang Z. Luan J. Zhang X. Liao B. (2023). Biomechanical analysis of lower limbs during stand-to-sit tasks in patients with early-stage knee osteoarthritis. Front. Bioeng. Biotechnol. 11, 1330082. 10.3389/fbioe.2023.1330082 38173868 PMC10763667

[B18] Rashid S. A. Hussain M. E. Bhati P. Veqar Z. Parveen A. Amin I. (2022). Muscle activation patterns around knee following neuromuscular training in patients with knee osteoarthritis: secondary analysis of a randomized clinical trial. Arch. Physiother. 12 (1), 19. 10.1186/s40945-022-00140-7 35799287 PMC9264689

[B19] Salamanna F. Caravelli S. Marchese L. Carniato M. Vocale E. Gardini G. (2023). Proprioception and mechanoreceptors in osteoarthritis: a systematic literature review. J. Clin. Med. 12 (20), 6623. 10.3390/jcm12206623 37892761 PMC10607296

[B20] Shanahan C. J. Hodges P. W. Wrigley T. V. Bennell K. L. Farrell M. J. (2015). Organisation of the motor cortex differs between people with and without knee osteoarthritis. Arthritis Res. Ther. 17 (1), 164. 10.1186/s13075-015-0676-4 26080802 PMC4494800

[B21] Smith J. A. Tain R. Sharp K. G. Glynn L. M. Van Dillen L. R. Henslee K. (2023). Identifying the neural correlates of anticipatory postural control: a novel fMRI paradigm. Hum. Brain Mapp. 44 (10), 4088–4100. 10.1002/hbm.26332 37162423 PMC10258523

[B22] Sohn R. Jenei-Lanzl Z. (2023). Role of the sympathetic nervous system in mild chronic inflammatory diseases: focus on osteoarthritis. Neuroimmunomodulation 30 (1), 143–166. 10.1159/000531798 37429263 PMC10428144

[B23] Soni A. Wanigasekera V. Mezue M. Cooper C. Javaid M. K. Price A. J. (2019). Central sensitization in knee osteoarthritis: relating presurgical brainstem neuroimaging and PainDETECT-Based patient stratification to arthroplasty outcome. Arthritis Rheumatol. 71 (4), 550–560. 10.1002/art.40749 30295432 PMC6430421

[B24] Strong A. Grip H. Boraxbekk C. J. Selling J. Häger C. K. (2022). Brain response to a knee proprioception task among persons with anterior cruciate ligament reconstruction and controls. Front. Hum. Neurosci. 16, 841874. 10.3389/fnhum.2022.841874 35392122 PMC8980265

[B25] Tang Y. Wang Z. Cao J. Tu Y. (2025). Bone-brain crosstalk in osteoarthritis: pathophysiology and interventions. Trends Mol. Med. 31 (3), 281–295. 10.1016/j.molmed.2024.09.006 39438197

[B26] Taniguchi M. Umehara J. Yamagata M. Yagi M. Motomura Y. Okada S. (2024). Understanding muscle coordination during gait based on muscle synergy and its association with symptoms in patients with knee osteoarthritis. Clin. Rheumatol. 43 (2), 743–752. 10.1007/s10067-023-06852-w 38133793

[B27] Yamauchi K. Suzuki S. Kato C. Kato T. (2020). Atrophy of individual thigh muscles measured by MRI in older adults with knee osteoarthritis: a cross-sectional study. Ann. Phys. Rehabil. Med. 63 (1), 38–45. 10.1016/j.rehab.2019.06.018 31386911

[B28] Yan C. G. Wang X. D. Zuo X. N. Zang Y. F. (2016). DPABI: data processing and analysis for (Resting-State) brain imaging. Neuroinformatics 14 (3), 339–351. 10.1007/s12021-016-9299-4 27075850

[B29] Yang M. P. Hou Y. Zeng X. L. (2024). Knee osteoarthritis and regularization. Chin. J. Jt. Surg. Electron. Ed. 18 (6), 497–504.

[B30] Zang Y. F. He Y. Zhu C. Z. Cao Q. J. Sui M. Q. Liang M. (2007). Altered baseline brain activity in children with ADHD revealed by resting-state functional MRI. Brain Dev. 29 (2), 83–91. 10.1016/j.braindev.2006.07.002 16919409

[B31] Zeng Z. Shan J. Zhang Y. Wang Y. Li C. Li J. (2022). Asymmetries and relationships between muscle strength, proprioception, biomechanics, and postural stability in patients with unilateral knee osteoarthritis. Front. Bioeng. Biotechnol. 10, 922832. 10.3389/fbioe.2022.922832 36185430 PMC9523444

[B32] Zou Q. H. Zhu C. Z. Yang Y. (2008). An improved approach to detection of amplitude of low-frequency fluctuation (ALFF) for resting-state fMRI: fractional ALFF. J. Neurosci. Methods 172 (1), 137–141. 10.1016/j.jneumeth 18501969 PMC3902859

[B33] Zuo X. N. Ehmke R. Mennes M. Imperati D. Castellanos F. X. Sporns O. (2012). Network centrality in the human functional connectome. Cereb. Cortex 22 (8), 1862–1875. 10.1093/cercor/bhr269 21968567

