# Supplementary material for: An exploratory study of peripheral joint deterioration and central sensorimotor network reorganization in people with knee osteoarthritis
Source: Front Bioeng Biotechnol. 2026 Oct 20;14:1898313. doi: 10.3389/fbioe.2026.1898313 (PMC13728353; doi:10.3389/fbioe.2026.1898313)
Supplement: Supplementary file 1 [file Supplementaryfile1.docx]

**Supplementary Material**

***Surface Electromyography (sEMG) Data of the Quadriceps Muscle***

**(1) Equipment and Experimental Protocol**

sEMG signals were acquired using an eight-channel wireless EMG system (Runyi Taiyi Technology, Shenzhen, China) with a sampling rate of 2000 Hz. Prior to electrode placement, the skin overlying the bilateral vastus lateralis (VL), rectus femoris (RF), and vastus medialis (VM) was shaved, abraded, and cleansed with 70% isopropyl alcohol to minimize impedance. Then disposable electrodes for the VL were placed at the motor point along the line connecting the anterior superior iliac spine (ASIS) and the lateral patella border. RF electrodes were affixed at the midpoint of the line between the ASIS and the superior patellar pole. VM electrodes were situated at the distal one-fifth of the line connecting the medial patella border and the ASIS. All electrodes were aligned parallel to the presumed direction of the muscle fibers.

**(2) Treadmill Walking Test**

Participants stood comfortably on a treadmill while the speed was gradually adjusted to a self-selected, comfortable pace ranging from 1 to 3 km/h. Following a 10-second acclimatization period, data were recorded for 15 consecutive gait cycles. Kinematic data were captured synchronously using the Opti_Knee motion analysis system (Innomotion Inc., Shanghai, China). Rigid plates, each equipped with four reflective markers, were secured 10 cm proximal and distal to the patella. A dual-lens stereoscopic infrared camera system tracked the three-dimensional trajectories of these markers at 60 Hz (accuracy: 0.3 mm RMS). Gait cycles were identified based on the trajectory data, and the stance phase (0%–60% of the gait cycle) was isolated for subsequent analysis.

**(3) Stair Ambulation Task**

Participants initiated the task from a stationary standing position upon an auditory cue. For stair ascent, participants led with the right limb, followed by the left, ascending three steps in total before pausing for one second on the third step. Stair descent followed the same limb sequence (right lead). The task was repeated three times to ensure reliability. Throughout the procedure, participants maintained an upright posture. sEMG and kinematic data were synchronized via a hardware trigger to ensure temporal alignment.

**(4) sEMG Signal Processing**

MATLAB analysis software was employed to process the original sEMG signals from the medial femoris and lateral femoris. The original electromyography signal is subjected to denoising via a 20–450 Hz bandpass Butterworth filter, followed by rectification and smoothing for preprocessing. The phase for walking we analyzed were Support phase of the gait cycle (0%–60%). This phase are significant because they correspond to critical phases of the gait cycle, each reflecting different biomechanical demands and kinematic behaviors. In the task of ascending and descending stairs, the limb that initiates the movement on the stairs is defined as the leading phase, while the limb that follows subsequently is referred to as the trailing phase. MATLAB analysis software was then employed to derive the amplitude values for all exercise cycles, determine the time range of each signal cycle based on the raw signals, and identify the Root Mean Square (RMS) and the Median

Frequency (MDF) for each muscle within the Support phase of the gait cycle (0%–60%) and leading phase. Additionally, the RMS were normalized [RMS(walking or up stair)/RMS(MVC)] using data from the MVC test.
